# Visualization of ^57^Fe-Labeled Heme Isotopic Fine Structure and Localization of Regions of Erythroblast Maturation in Mouse Spleen by MALDI FTICR-MS Imaging

**DOI:** 10.1007/s13361-017-1768-y

**Published:** 2017-08-17

**Authors:** Makoto Kihara, Yukari Matsuo-Tezuka, Mariko Noguchi-Sasaki, Keigo Yorozu, Mitsue Kurasawa, Yasushi Shimonaka, Michinori Hirata

**Affiliations:** Product Research Department, Medical Affairs Division, Chugai Pharmaceutical Co. Ltd., 200 Kajiwara, Kamakura, Kanagawa 247-8530 Japan

**Keywords:** MALDI FTICR-MS imaging, Isotopic fine structure, High resolution, Heme, Epoetin beta pegol

## Abstract

Epoetin beta pegol (continuous erythropoiesis receptor activator; C.E.R.A.), or methoxy-polyethylene glycol-modified epoetin beta, is a long-acting erythropoiesis stimulating agent (ESA) that effectively maintains hemoglobin levels. It promotes proliferation of erythroid progenitor cells in hematopoietic organs and leads to increased reticulocyte and hemoglobin levels. However, the detailed erythropoietic effects of various ESAs on their target organs have yet to be clarified, and new approaches are needed to analyze tissue iron localization with structural information. Matrix-assisted laser desorption/ionization (MALDI) mass spectrometry imaging (MSI) techniques are widely used in basic pharmaceutical research. High-resolution Fourier transform ion cyclotron resonance (FTICR) mass spectrometry (MS) imaging enables the spatial mapping and identification of biomolecules. In this study, mice administered with C.E.R.A. were fed a diet containing the stable iron isotope ^57^Fe. The ^57^Fe-heme^+^ isotopic fine structure peak (*m/z* 617.1772) was separated from the non-labeled heme^+^ isotopic peak (Δ0.0029) by FTICR-MS with a resolving power of more than 500,000. We optimized the platform to analyze the distribution of ^57^Fe-heme in the spleen using MALDI FTICR-MS imaging. The combination of the ultrahigh resolution power of FTICR-MS and a stable isotope labeling technique has the potential to be very effective in basic pharmaceutical research.

**Electronic supplementary material:**

The online version of this article (doi:10.1007/s13361-017-1768-y) contains supplementary material, which is available to authorized users.

**Graphical Abstract:**
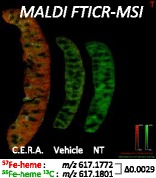
ᅟ

## Introduction

Erythropoietin is one of several hematopoietic factors and is essential for erythrocyte production. Erythropoiesis stimulating agents (ESAs), the active ingredients of whichare recombinant human erythropoietin or its modified form, have been widely used to treat patients with certain kinds of anemia, for example chronic kidney disease-associated anemia, in clinical practice [1]. In clinical settings, several ESA preparations are now available. C.E.R.A. is one of the long-acting ESAs. It differs from epoetin beta in having chemically attached methoxy-polyethylene glycol, which leads to extremely prolonged blood retention [2, 3]. Although there are differences among ESAs in terms of receptor binding affinity and blood retention, for example, the differences in erythropoietic effects in their target organs, such as bone marrow and spleen, have not yet been clarified.

Natural iron is composed of four stable iron isotopes, ^54^Fe, ^56^Fe, ^57^Fe, and ^58^Fe, the relative isotopic abundances of which are 5.845%, 91.754%, 2.119%, and 0.282%, respectively [4, 5]. Instead of radioactive isotopes, ^57^Fe has been used as a tracer of exogenous iron in nutritional studies because of its low natural abundance, and inductively coupled plasma mass spectrometry (ICP-MS) for determination of stable iron isotopes ensures high sensitivity and provides quantitative data. ^57^Fe and ICP-MS have already been used to assess the process of iron absorption [5, 6]. Our recent study, combining ^57^Fe-labeling with ICP-MS technology, has provided quantitative analysis of dietary iron utilization for hemoglobin synthesis under stress erythropoiesis induced by C.E.R.A. administration in mice [7]. Based on the analysis of ^57^Fe-iron incorporation by ICP-MS, we were able to quantitatively observe not only the dynamics of dietary iron utilization for hemoglobin synthesis but also dietary iron distribution among tissues. Although this platform is very useful for analyzing the iron uptake, utilization, distribution and accumulation in tissues, and trafficking across tissues, another approach is necessary to analyze detailed tissue iron localization with structural information.

Fourier transform ion cyclotron resonance mass spectrometry (FTICR-MS) is used to analyze extremely complex mixtures such as crude oil [8], proteome [9], and metabolome samples [10] because of its ultrahigh resolution (>1,000,000 full width at half-maximum at 7.0 T) and high mass accuracy (<1 ppm). Ultrahigh resolution mass spectrometry is also used to determine unknown compounds [11]. The ultrahigh resolution of FTICR-MS enable clear separation of stable isotopes such as ^13^C, ^15^N, ^34^S, and ^18^O, and the high mass accuracy allows candidates elemental compositions [12].

Matrix-assisted laser desorption/ionization (MALDI) mass spectrometry imaging (MSI) techniques are widely used in basic research targeting biomolecules such as lipids, peptides, and proteins. The MALDI MSI technique has been used to image drug molecule and metabolite distribution in tissue sections [13–15]. Heme, as a cofactor of hemoglobin in red blood cells, is applicable for visualization target of the lumen of blood vessels in tissue [15].

MSI applications are increasingly providing more detailed visualization of administered drugs and localization of their metabolites in the tissues. In the case of MALDI-time-of-flight (TOF) MSI, the integration of rapid data acquisition, narrow laser focus (5–20 μm), and development of image analytical software established the capacity of producing high-resolution 3D images [16, 17]. These detailed 3D images were expected to have applications in biomarker discovery and in diagnosis [17]. However, the number of studies on potential applications of MALDI FTICR-MSI is still limited. Recently, Spraggins et al. reported the use of MALDI FTICR-MSI for protein analysis [18]. In their study, the ultrahigh resolution power and high mass accuracy (<5 ppm) of FTICR-MS enabled proteins to be identified with high levels of confidence [18]. The results indicated that FTICR-MS ultrahigh resolution power may be applicable to a MSI technique for biological molecules. The combination of FTICR-MS ultrahigh resolution power with a stable isotope labeling technique offers the potential of identifying the isotopic fine structure of specific biological molecules and visualize their localization in tissues. Here, we have demonstrated the application of MALDI FTICR-MSI combined with stable isotope labeling in pharmaceutical basic research. MALDI FTICR-MSI offers a solution to the problem of visualizing the fine isotopic structure of ^57^Fe-heme in mouse hematopoietic organs.

## Experimental

### Materials

C.E.R.A. used in this study was produced by Chugai Pharmaceutical Co., Ltd. (Tokyo, Japan). Stable iron isotope ^57^Fe powder (95.95% purity) was purchased from Medical Isotopes (Pelham, NH, USA). All diets used in this study were purchased from Research Diets (New Brunswick, NJ, USA). This study used the following diets: ^57^Fe diet (diet mixed with 200 ppm of ^57^Fe instead of natural iron); and control diet (diet mixed with 200 ppm of natural iron). Male C57BL/6NCrl mice were purchased from Charles River Laboratories Japan (Hino, Japan). All animals were allowed to acclimatize and recover from shipping-related stress for 4 to 6 d prior to the study. Mice were housed under specific pathogen-free conditions with free access to food and water. All studies were approved by the Institutional Animal Care and Use Committee at Chugai Pharmaceutical Co., Ltd. C.E.R.A. was diluted to appropriate concentrations in phosphate buffer vehicle (PBS containing 0.02% polyoxyethylene sorbitan monooleate [Tween 80]). To evaluate the incorporation of labeled iron during C.E.R.A. treatment, mice fed the control diet were switched to the ^57^Fe diet immediately after being intravenously administered 10 μg/kg of C.E.R.A. or vehicle in a single injection. On day 5 after administration, mice were euthanized by exsanguination under anesthesia with isoflurane. Two mouse spleens from each group were harvested for MALDI FTICR-MSI.

### Sample Preparation

The harvested spleens from each group (non-treatment [NT], vehicle, and C.E.R.A.) were fresh frozen with 2% carboxymethyl cellulose compounds. Frozen sections were cut at a thickness of 10 μm using a cryostat (CM3050S; Leica, Nussloch, Germany). The sections were coated with 30 mg/mL 2,5-dihydroxybenzoic acid (DHB; Wako Pure Chemical Industries, Ltd., Osaka, Japan) matrix solution (50% methanol, 0.1% TFA) using a TM-sprayer (HTX Technologies, Chapel Hill, NC, USA) without an ethanol wash. The matrix spraying conditions were velocity 1333 mm/min., flow rate 0.12 mL/min., temperature 110 °C, number of passes 4 (rotated 90° after pass #2), offsetting 1.5 mm, track spacing 3 mm, and sheath gas pressure 10 psi.

### Mass Spectrometry: Data Acquisition

MALDI MS analyses were performed on a 7T solariX FTICR-MS (Bruker Daltonics, Billerica, MA, USA) in positive ion mode. Transients of 2 mega-words were collected for an experimental mass resolving power (*m/Δm*_50%_) of more than 500,000 at *m/z* 616.1768. MS data were acquired with a single scan and step size of 50 μm with 98% data reduction mode between *m/z* 601.92 and 650.00 from 37,935 positions. The laser was operated at 1000 Hz with 150 laser shots at each position. The continuous accumulation of selected ions (CASI) was set as Q1 *m/z* 617.2 (isolation window: *m/z* 50.0) in FTMS control software (Bruker Daltonics). Lock mass calibration was performed at *m/z* 616.1768 in FTMS control software (Bruker Daltonics) [19]. MALDI MS images were generated using flexImaging software (Bruker Daltonics) with Δ*m/z* = ± 0.001.

## Results and Discussion

### Detection of Heme b Signals

**Figure 1:**
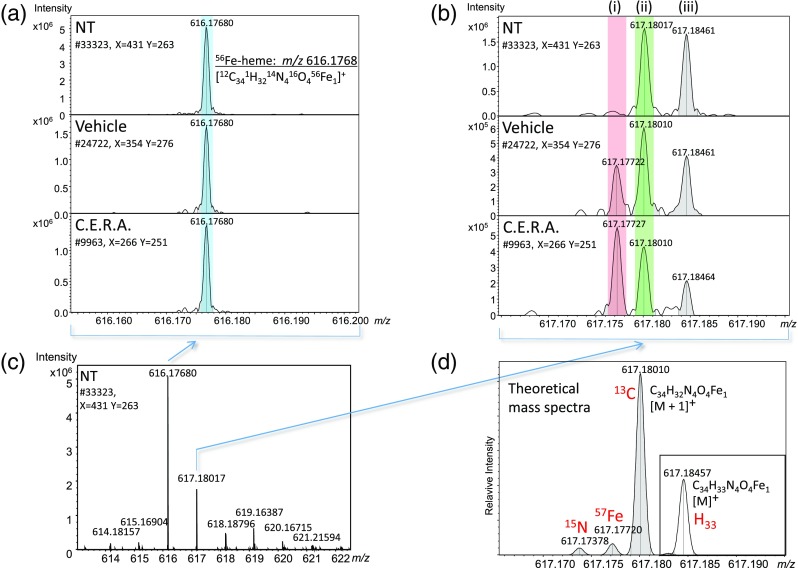
MALDI FTICR-MS spectra of heme b isotopic fine structure. (**a**) Expanded *m/z* window of heme b monoisotopic peak. These MS data were acquired at positions #33323/NT, #24722/vehicle, and #9963/C.E.R.A., shown in Figure 2e as white arrows. The MS data of heme b monoisotopic mass ([M]^+^), (*m/z* 616.1768 ± 0.001; pale blue line) was analyzed by flexImaging software and imaged as ^56^Fe-heme (Figure 2a). The ^56^Fe-heme peak resolving powers (RP) were 627,507 (#33323/NT), 649,350 (#24722/vehicle), and 639,973 (#9963/C.E.R.A.), respectively, at each position. The heme b monoisotopic mass *m/z* 616.1768 was set as the single point lock mass for MALDI FTICR-MSI. (**b**) Expanded *m/z* window of heme b first isotopic peak M + 1. The peaks, (i) ^57^Fe-heme: *m/z* 617.1772 [^12^C_34_^1^H_32_^14^N_4_^16^O_4_^57^Fe_1_]^+^, (ii) ^56^Fe-heme ^13^C: *m/z* 617.1801 [^12^C_33_^13^C_1_^1^H_32_^14^N_4_^16^O_4_^56^ Fe_1_]^+^, and (iii) ^56^Fe-heme H_33_: *m/z* 617.1846 [^12^C_34_^1^H_33_^14^N_4_^16^O_4_^56^Fe_1_]^+^, were well separated with more than 500,000 RP. ^57^Fe-heme (*m/z* 617.1772 ± 0.001; pale orange line), and ^56^Fe-heme ^13^C (*m/z* 617.1801 ± 0.001; pale green line) were imaged as ^57^Fe-heme and ^56^Fe-heme ^13^C, respectively, (Figure 2b, c). (**c**) The heme b isotope distribution pattern in the mass range of *m/z* 614–621 at position #33323/NT. (**d**) Theoretical isotopic distribution patterns at RP approximately 617,000. Theoretical mass spectra C_34_H_32_N_4_O_4_ Fe_1_ (M + 1) and C_34_H_33_N_4_O_4_ Fe_1_ (M) are merged

The heme b signals were efficiently observed from mouse spleens. A typical MALDI FTICR-MS spectrum is shown in Figure 1c. It was observed in the mass range *m/z* 614–621 from position #33,323 in non-treated mouse spleen. The observed ion isotopic pattern matched the theoretical isotopic distribution of heme b ions ([^12^C_32-34_^13^C_0-2_^1^H_31-33_^14^N_4_^16^O_4_^54-58^Fe_1_]^+^) [20] that was calculated with DataAnalysis software (Bruker Daltonics). These heme b isotopic ions were detected efficiently as [M]^+^ by MALDI FTICR-MS from mouse spleen sections. In more broad mass range measurement [M + K]^+^ adduct ions were also observed but the intensity was weaker than that of [M]^+^ (data not shown). Therefore, we focused on heme b [M]^+^ for further analysis. It is well known that MALDI ionization is suppressed by salts and some types of lipid, so we compared the effects of using the 70% ethanol wash method or Carnoy’s fluid wash method [21] as a pretreatment, or no pretreatment, before using the matrix spray. We found that the heme b MALDI FTICR-MS intensity gradient patterns were quite similar with or without pretreatment, but that the heme b MALDI FTICR-MS intensity levels were reduced with 70% ethanol or Carnoy’s fluid wash (data not shown). The volatile buffer (50 mM ammonium formate, etc.) wash has the possibility of enhancing signal intensity [22]. But target molecules like proteins tend to be leaked. We could not find a washing condition suitable for heme b MSI (data not shown). In our experiments, the pretreatment wash before the matrix spray was therefore not effective for heme ionization in mouse spleens.

### Detailed Analysis of Heme b First Isotopic Peak M + 1

In hematopoietic organs such as bone marrow and spleen, erythroid progenitors proliferate and differentiate into erythroblasts supported by erythropoietin [23]. Immature erythroblasts are induced to transcribe erythroid-specific 5-aminolevulinic acid synthase, the key enzyme of heme synthesis, which in turn triggers subsequent hemoglobin synthesis, leading to maturation of erythroblasts into erythrocytes [24]. As shown in our previous study [7], C.E.R.A. also stimulates hemoglobin synthesis partly using dietary iron, as assessed using an ^57^Fe-substituted diet and the ICP-MS platform.

The heme b first isotopic peak M + 1 was resolved using FTICR-MS with more than 500,000 resolving power, and extended FTICR-MS profiles were shown from different positions (#33323/NT, #24722/vehicle, #9963/C.E.R.A., Figure 1b). The isotopic fine structure of heme b peak M + 1, including ^57^Fe-heme [^12^C_34_^1^H_32_^14^N_4_^16^O_4_^57^Fe_1_]^+^ at *m/z* 617.1772, ^56^Fe-heme ^13^C [^12^C_33_^13^C_1_^1^H_32_^14^N_4_^16^O_4_^56^Fe_1_]^+^ at *m/z* 617.1801, and ^56^Fe-heme H_33_ [^12^C_34_^1^H_33_^14^N_4_^16^O_4_^56^Fe_1_]^+^ at *m/z* 617.1846, was observed. In the NT group, in which mice were fed the control diet, ^56^Fe-heme ^13^C and ^56^Fe-heme H_33_ peaks were detected but a ^57^Fe-heme peak was not identified within heme isotopic peak M + 1. However, in the vehicle and C.E.R.A.-treated groups, in which mice fed the control diet were switched to the ^57^Fe diet immediately after injection, ^57^Fe-heme peaks were detected in addition to ^56^Fe-heme ^13^C and ^56^Fe-heme H_33_ peaks (Figure 1b). Overall average spectra from 37,935 data positions were calculated with flexImaging software, and the ^57^Fe-heme peak (*m/z* 617.1772), ^56^Fe-heme ^13^C peak (*m/z* 617.1801), and ^56^Fe-heme H_33_ peak (*m/z* 617.1846) were well separated (data not shown).

### MALDI FTICR-MSI of ^57^Fe-Heme and Non-Labeled Heme b Isotopes

**Figure 2:**
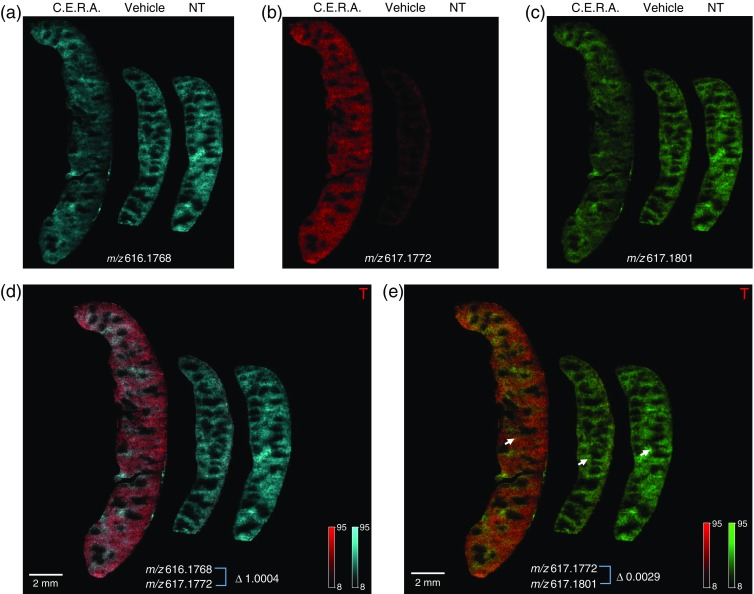
MALDI FTICR-MSI for ^57^Fe-heme and ^56^Fe-heme isotopes in mouse spleens. MSI data from (**a**) ^56^Fe-heme monoisotopic mass (^56^Fe-heme, *m/z* 616.1768 ± 0.001; pale blue line); (**b**) ^57^Fe-heme (*m/z* 617.1772 ± 0.001; pale orange line); and (**c**) ^56^Fe-heme ^13^C (*m/z* 617.1801 ± 0.001; pale green line) in C.E.R.A.-treated, vehicle-treated, and NT mouse spleens. In C.E.R.A.-treated mouse spleen, newly synthesized ^57^Fe-heme (*m/z* 617.1772) derived from the ^57^Fe-diet was clearly detectable, in contrast to vehicle-treated mouse spleen (**b**). (**d**) Merged MSI data from ^56^Fe-heme monoisotopic mass (**a**) and ^57^Fe-heme (**b**) (Δ1.0004). (**e**) Merged MSI data from ^56^Fe-heme ^13^C (**c**) and ^57^Fe-heme (**b**) (Δ0.0029). Scale bar indicates 2 mm (**d**), (**e**)

In our experiment, splenomegaly (enlargement of the spleen) was observed in the C.E.R.A.-treated group (Figure 2). In rodents under stress erythropoiesis, extramedullary hematopoiesis is induced and splenomegaly is often observed because of the expansion of erythroid lineage cells. As previously reported [7], C.E.R.A. also induced extramedullary hematopoiesis accompanied by splenomegaly. Furthermore, the spleen, a component of the reticuloendothelium, is also known to act as an iron store, in which iron is stored in its ferritin-bound form. For these reasons, we selected spleen to visualize ^57^Fe-heme localization.

In FTICR-MSI to resolve isotopic fine structure peaks, precise mass calibration is critical at every data acquisition point. We have optimized a MALDI FTICR-MSI platform in which data acquisition of the heme b monoisotopic mass (^56^Fe-heme) *m/z* 616.1768 was set as the single point lock mass, and accurate *m/z* adjustment was performed through all data points [19]. FlexImaging software visualized the ^57^Fe-heme peak (*m/z* 617.1772 ± 0.001; red) and the ^56^Fe-heme ^13^C peak (*m/z* 617.1801 ± 0.001; green) based on their peak intensities and location information (Figure 2).

The ^57^Fe-heme peak (*m/z* 617.1772 ± 0.001; red) was detected in the entire spleen area with signal intensity gradients in C.E.R.A.-treated mice, detected at very low levels in vehicle-treated mice, and was almost undetectable in NT mice (Figure 2b). The ^56^Fe-heme ^13^C peak (*m/z* 617.1801 ± 0.001; green) was detected in the entire spleen area with signal intensity gradients in C.E.R.A.-treated, vehicle-treated, and NT mice (Figure 2c). There was a Δ0.0029 difference between the ^57^Fe-heme peak and the ^56^Fe-heme ^13^C peak (*m/z* 617.1801). The heme b monoisotopic mass peak (^56^Fe-heme, *m/z* 616.1768 ± 0.001; pale blue) was visualized and detected in the entire spleen area in C.E.R.A.-treated, vehicle-treated, and NT mice (Figure 2a). The MSI results for the heme b monoisotopic mass peak (^56^Fe-heme) showed similarity to the signal intensity gradient pattern of the ^56^Fe-heme ^13^C peak (*m/z* 617.1801 ± 0.001; green; Figure 2c). Overlay of the ^57^Fe-heme peak (*m/z* 617.1772 ± 0.001; red) and ^56^Fe-heme ^13^C peak (*m/z* 617.1801 ± 0.001; green) was used to visualize the relative intensity of heme ions, with color gradation from red (derived from newly synthesized heme) to yellow to green (derived from non-labeled heme) in the spleen (Figure 2e). In the same way, overlay of the ^57^Fe-heme peak (*m/z* 617.1772 ± 0.001; red) and heme b monoisotopic mass peak (^56^Fe-heme, *m/z* 616.1768 ± 0.001; pale blue) was also used to visualize the relative intensity of heme ions, with color gradation from red (derived from newly synthesized heme) to white to pale blue (derived from non-labeled heme) (Figure 2d). There was a Δ1.0004 difference between the ^57^Fe-heme peak (*m/z* 617.1772) and the heme b monoisotopic mass peak *m/z* 616.1768, which was fixed as a lock mass.

Comparing the overlay image results in Figure 2d and e, the relative intensities of [^57^Fe-heme/^56^Fe-heme] and [^57^Fe-heme/^56^Fe -heme ^13^C] showed exactly the same distribution of gradient patterns in mouse spleens. This result strongly confirms the precision of all acquired data from 37,935 mouse spleen positions with mass resolving power >500,000. The mass accuracies were better than 1.6 ppm between *m/z* 601.92 and 650.00. Our optimized MALDI FTICR-MSI platform was able to resolve heme M + 1 isotopic fine structure and distinguish the ^57^Fe-labeled heme peak from the non-labeled ^56^Fe-heme ^13^C peak with a Δ0.0029 difference.

### Histologic Validation

**Figure 3:**
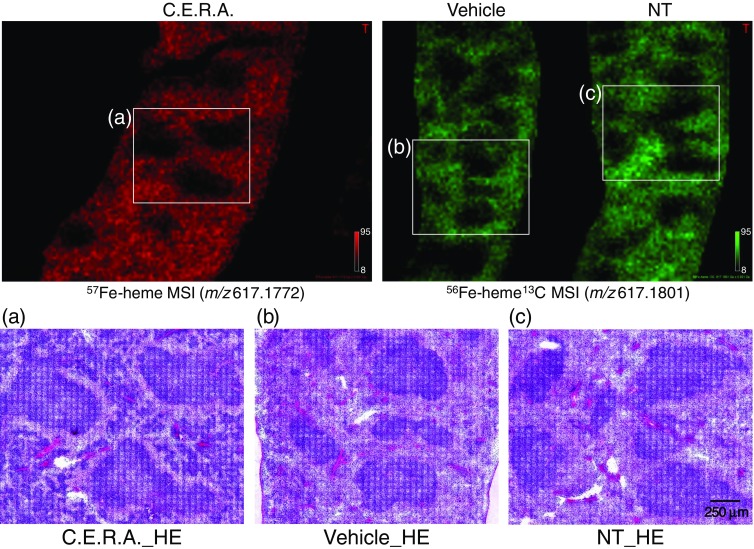
H&E staining of mouse spleens. Sections of the MS images shown in Figure 2b and c are expanded and compared with the same areas stained with H&E (**a**), (**b**), (**c**). The white pulp (lymphocyte area) is located in areas with extremely low signal in the MS images. The red pulp (erythrocyte area) is located in areas with a strong signal. In C.E.R.A.-treated spleen, increased numbers of erythroblasts are observed (**a**). Scale bar indicates 250 μm (Figure 3)

Following MALDI MSI data acquisition, the DHB matrix was removed from the sample slides by washing with 70% ethanol. The slides were stained with H&E and histologic evaluation was performed to observe the red pulp and white pulp structures of the spleen (Figure 3). In the NT and vehicle-treated mouse spleens, there was an obvious match between the localization pattern of non-labeled heme (^56^Fe-heme and ^56^Fe-heme ^13^C) ion signal intensity from the MSI results and the pattern of red pulp on histology. The areas where ^56^Fe-heme ion intensities were extremely low in the MSI results corresponded with the white pulp (Figure 3b, c). The data positions #33323/NT, #24722/vehicle, and #9963/C.E.R.A. in Figure 1 were located in red pulp in mouse spleens. In C.E.R.A.-treated mouse spleens, there was a noticeable increase in hematopoietic cells (extramedullary hematopoiesis) (Figure 3a). These results strongly correlated with the localization pattern of ^57^Fe-heme ion intensity in the MSI results. ^57^Fe-heme ions were weakly detected in the red pulp area of vehicle-treated mouse spleens (Figure 2b). It is assumed that the stable iron isotope ^57^Fe from the diet was incorporated into porphyrin in this area. The difference in ^57^Fe-heme ion intensity between vehicle and C.E.R.A.-treated mice indicates the mode of action of C.E.R.A. and its drug efficacy in mouse spleens.

In H&E staining images, scars of laser shots (150 shots/pixel) from MALDI MSI data acquisitions were clearly observed (Figure 3). These images displayed evidence that all data were acquired with a precise 50 μm pitch from 37,935 mouse spleen positions, and that the diameter of the laser-pulsed field was less than 50 μm/pixel. These results proved the reliability of the MALDI FTICR-MSI platform in which noise signals from adjacent pixels were minimized.

## Conclusions

Our recent study, combining ^57^Fe-labeling with ICP-MS technology, provided quantitative analysis of dietary iron utilization for hemoglobin synthesis. However, another approach was necessary to analyze detailed tissue iron localization with structural information.

In this report, we successfully and completely separated and specifically detected ^57^Fe-labeled heme isotopic structure from other non-labeled heme peaks using FTICR-MS with ultrahigh resolution power. As a result of effective ionization of heme b and improvement of the lock mass setting, a MALDI FTICR-MSI platform was optimized. The distribution of newly synthesized ^57^Fe-heme in C.E.R.A.-treated mouse spleens was visualized using the MALDI FTICR-MSI platform. Histologic validation by H&E staining demonstrated the expansion of erythroblasts in C.E.R.A.-treated mouse spleens, confirming the MSI results. These results strongly indicate the effectiveness and reliability of the MALDI FTICR-MSI platform we have optimized. Heme b is a key molecule in vivo, and it is also an indicator of C.E.R.A. drug efficacy. This is the reason why we focused on the heme b isotopes for this investigation. We conclude that the combination of ultrahigh resolution FTICR-MS and stable isotope labeling techniques will be very effective in basic pharmaceutical research. The trend of MALDI MSI is rapid data acquisition to build high resolution 3D images, but on the other hand, MALDI FTICR-MSI platform might be another solution.

## Electronic supplementary material

Below is the link to the electronic supplementary material.

ESM 1 (PDF 597 kb)
