# Supplementary material for: Visualization of 57Fe-Labeled Heme Isotopic Fine Structure and Localization of Regions of Erythroblast Maturation in Mouse Spleen by MALDI FTICR-MS Imaging
Source: J Am Soc Mass Spectrom. 2017 Aug 17;28(11):2469–75. doi: 10.1007/s13361-017-1768-y (PMC5645437; doi:10.1007/s13361-017-1768-y)
Supplement: Supplementary file 1 — (PDF 597 kb) [file 13361_2017_1768_MOESM1_ESM.pdf]

Figure 2

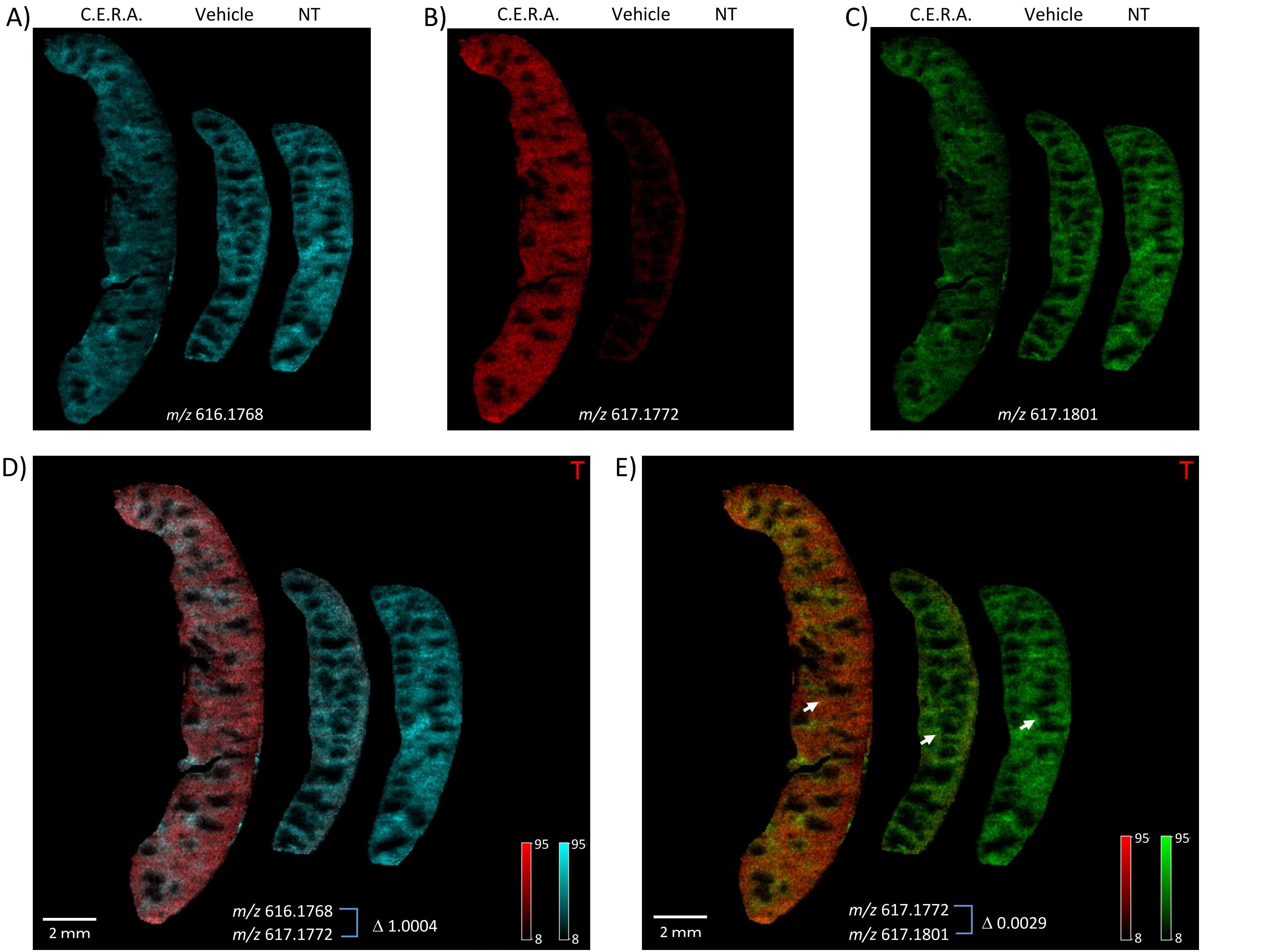

A)
C.E.R.A.
Vehicle
NT
m/z 616.1768
B)
C.E.R.A.
Vehicle
NT
m/z 617.1772
C)
C.E.R.A.
Vehicle
NT
m/z 617.1801
D)
T
m/z 616.1768
m/z 617.1772
Δ 1.0004
95
8
95
8
2 mm
E)
T
m/z 617.1772
m/z 617.1801
Δ 0.0029
95
8
95
8
2 mm

The MSI result of 50 mM Ammonium formate pH 6.4 wash for reviewing

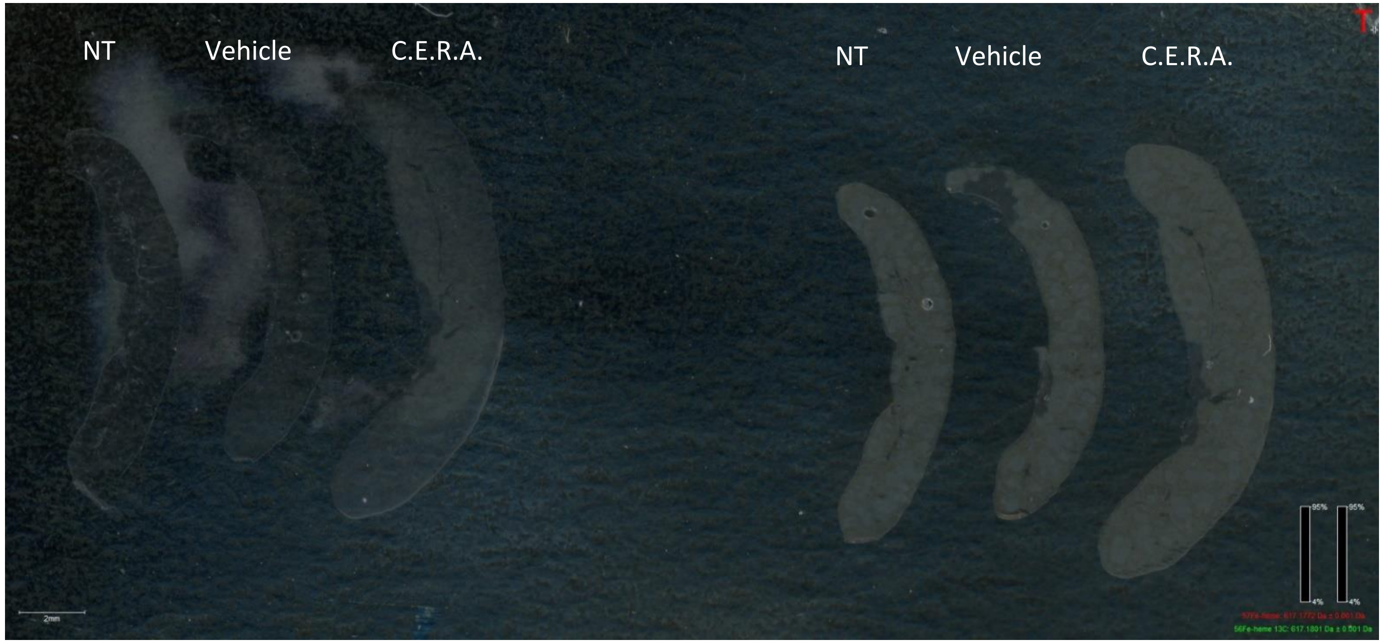

Slide：heme Day 5 spleens

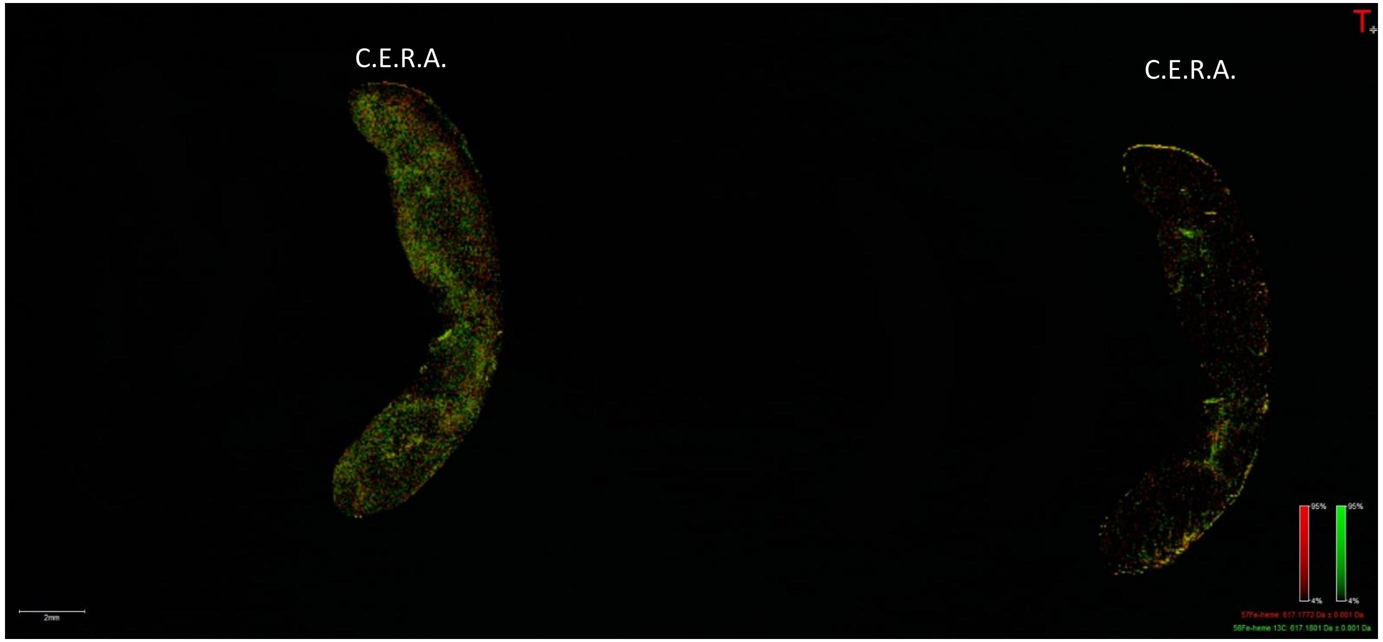

with Ammonium formate wash

w/o wash

MALDI FTICR-MSI result;
with / without AF wash
